# Viral Antigen and Inflammatory Biomarkers in Cerebrospinal Fluid in Patients With COVID-19 Infection and Neurologic Symptoms Compared With Control Participants Without Infection or Neurologic Symptoms

**DOI:** 10.1001/jamanetworkopen.2022.13253

**Published:** 2022-05-23

**Authors:** Arvid Edén, Anna Grahn, Daniel Bremell, Anahit Aghvanyan, Pradeepthi Bathala, Dietmar Fuchs, Johanna Gostner, Lars Hagberg, Nelly Kanberg, Sunsanee Kanjananimmanont, Magnus Lindh, Salvia Misaghian, Staffan Nilsson, Michael Schöll, George Sigal, Erika Stentoft, Marie Studahl, Aylin Yilmaz, Mingyue Wang, Martin Stengelin, Henrik Zetterberg, Magnus Gisslén

**Affiliations:** 1 Department of Infectious Diseases, Institute of Biomedicine, Sahlgrenska Academy, University of Gothenburg, Gothenburg, Sweden; 2 Region Västra Götaland, Department of Infectious Diseases, Sahlgrenska University Hospital, Gothenburg, Sweden; 3 Meso Scale Diagnostics LLC, Rockville, Maryland; 4 Institute of Biological Chemistry, Medical University of Innsbruck, Biocenter, Austria; 5 Institute of Medical Biochemistry, Medical University of Innsbruck, Biocenter, Austria; 6 Department of Laboratory Medicine, Institute of Biomedicine, Sahlgrenska Academy, University of Gothenburg, Gothenburg, Sweden; 7 Department of Psychiatry and Neurochemistry, Institute of Neuroscience and Physiology, The Sahlgrenska Academy at the University of Gothenburg, Gothenburg, Sweden; 8 Wallenberg Centre for Molecular and Translational Medicine, University of Gothenburg, Gothenburg, Sweden; 9 Department of Neurodegenerative Disease, UCL Institute of Neurology, London, England; 10 Clinical Neurochemistry Laboratory, Sahlgrenska University Hospital, Mölndal, Sweden; 11 UK Dementia Research Institute at UCL, London, England; 12 Hong Kong Center for Neurodegenerative Diseases, Clear Water Bay, Hong Kong, China

## Abstract

**Importance:**

Neurologic symptoms are common in COVID-19, but the central nervous system (CNS) pathogenesis is unclear, and viral RNA is rarely detected in cerebrospinal fluid (CSF).

**Objective:**

To measure viral antigen and inflammatory biomarkers in CSF in relation to neurologic symptoms and disease severity.

**Design, Setting, and Participants:**

This cross-sectional study was performed from March 1, 2020, to June 30, 2021, in patients 18 years or older who were admitted to Sahlgrenska University Hospital, Gothenburg, Sweden, with COVID-19. All patients had CSF samples taken because of neurologic symptoms or within a study protocol. Healthy volunteer and prepandemic control groups were included.

**Exposure:**

SARS-CoV-2 infection.

**Main Outcomes and Measures:**

Outcomes included CSF SARS-CoV-2 nucleocapsid antigen (N-Ag) using an ultrasensitive antigen capture immunoassay platform and CSF biomarkers of immune activation (neopterin, β^2^-microglobulin, and cytokines) and neuronal injury (neurofilament light protein [NfL]).

**Results:**

Forty-four patients (median [IQR] age, 57 [48-69] years; 30 [68%] male; 26 with moderate COVID-19 and 18 with severe COVID-19 based on the World Health Organization Clinical Progression Scale), 10 healthy controls (median [IQR] age, 58 [54-60] years; 5 [50%] male), and 41 patient controls (COVID negative without evidence of CNS infection) (median [IQR] age, 59 [49-70] years; 19 [46%] male) were included in the study. Twenty-one patients were neuroasymptomatic and 23 were neurosymptomatic (21 with encephalopathy). In 31 of 35 patients for whom data were available (89%), CSF N-Ag was detected; viral RNA test results were negative in all. Nucleocapsid antigen was significantly correlated with CSF neopterin (*r* = 0.38; *P* = .03) and interferon γ (*r* = 0.42; *P* = .01). No differences in CSF N-Ag concentrations were found between patient groups. Patients had markedly increased CSF neopterin, β_2_-microglobulin, interleukin (IL) 2, IL-6, IL-10, and tumor necrosis factor α compared with controls. Neurosymptomatic patients had significantly higher median (IQR) CSF interferon γ (86 [47-172] vs 21 [17-81] fg/mL; *P* = .03) and had a significantly higher inflammatory biomarker profile using principal component analysis compared with neuroasymptomatic patients (0.54; 95% CI, 0.03-1.05; *P* = .04). Age-adjusted median (IQR) CSF NfL concentrations were higher in patients compared with controls (960 [673-1307] vs 618 [489-786] ng/L; *P* = .002). No differences were seen in any CSF biomarkers in moderate compared with severe disease.

**Conclusions and Relevance:**

In this study of Swedish adults with COVID-19 infection and neurologic symptoms, compared with control participants, viral antigen was detectable in CSF and correlated with CNS immune activation. Patients with COVID-19 had signs of neuroaxonal injury, and neurosymptomatic patients had a more marked inflammatory profile that could not be attributed to differences in COVID-19 severity. These results highlight the clinical relevance of neurologic symptoms and suggest that viral components can contribute to CNS immune responses without direct viral invasion.

## Key Points

**Question:**

Are cerebrospinal fluid (CSF) SARS-CoV-2 antigens associated with central nervous system inflammation in patients with COVID-19?

**Findings:**

Of 44 patients with COVID-19 (23 neurosymptomatic) included in this hospital-based cross-sectional study, CSF nucleocapsid antigen was detectable in 89% of patients with available data and was significantly correlated with immune activation markers (neopterin and interferon γ). Moreover, neurosymptomatic patients had a more pronounced inflammatory CSF profile compared with neuroasymptomatic patients that could not be attributed to differences in COVID-19 severity.

**Meaning:**

These results suggest that viral components may contribute to central nervous system immune responses without direct viral invasion and highlight the clinical importance of neurologic symptoms.

## Teaser

This cross-sectional study assesses whether viral antigens are detectable in the cerebrospinal fluid of patients with COVID-19 and compares cerebrospinal fluid biomarker profiles of patients with and without neurologic symptoms and with moderate vs severe COVID-19.

## Introduction

Neurologic symptoms are common in patients hospitalized with COVID-19 and include anosmia, encephalopathy, encephalitis, and cerebrovascular manifestations, including stroke.^1,2,3^ Several mechanisms have been proposed to contribute to the central nervous system (CNS) pathogenesis, including direct damage caused by viral CNS invasion, direct or indirect consequences of the systemic inflammatory response, viral interaction with the neurovascular unit or microvascular injuries, and thromboembolic events.^4,5^

The neuroinvasive capacity of SARS-CoV-2 is questionable. Although virus has been suggested to enter the CNS via olfactory sensory neurons, neuropathologic studies^6,7^ have shown conflicting results. Furthermore, viral detection in brain tissue is often sporadic, of low levels, and not clearly linked to neuropathologic signs of infection.^8^ In cerebrospinal fluid (CSF), viral RNA can only be detected at very low levels in a few cases, and characteristic signs of neurotropic viral infections (CSF pleocytosis and blood-brain barrier injury) are often mild or absent.^4,9^ Although viral RNA is rarely detected, intrathecal immune activation has been a distinguishing feature in patients with neurologic symptoms.^10,11^ Biomarker signs of neuroaxonal injury have also been observed, most evident in patients with encephalitis or stroke.^10,12,13,14^

The cause of the CSF immune response in COVID-19 in the absence of detectable viral RNA is not clear. SARS-CoV-2 viral nucleocapsid antigen (N-Ag) can be detected in plasma during the acute phase of infection and is potentially useful as a diagnostic as well as prognostic marker in COVID-19.^15^ However, little is known about the presence of viral antigens in CSF. The aims of this study were to investigate whether viral antigens could be detected in CSF in patients with COVID-19 and to compare CSF biomarker profiles of patients with or without neurologic symptoms as well as in patients with moderate compared with severe COVID-19.

## Methods

### Study Population, Procedures, and Viral Diagnostics

This prospective cross-sectional study included adult (≥18 years of age) patients with confirmed SARS-CoV-2 infection admitted to Sahlgrenska University Hospital in Gothenburg, Sweden, who had undergone a diagnostic lumbar puncture because of neurologic symptoms or as part of a research protocol. This study was performed from March 1, 2020, to June 30, 2021. All participants (or next of kin if the patient was not able) provided written informed consent according to the Declaration of Helsinki.^16^ The study protocol was approved by the Swedish Ethical Review Authority. The report follows the Strengthening the Reporting of Observational Studies in Epidemiology (STROBE) reporting guideline for cross-sectional studies.

SARS-CoV-2 infection was confirmed by viral RNA detection in real-time polymerase chain reaction assays of nasopharyngeal swab or plasma (n = 1) specimens or in-hospital seroconversion (n = 1). SARS-CoV-2 RNA was analyzed by real-time polymerase chain reaction in swab specimens and cell-free CSF and plasma samples as previously described (eMethods in the Supplement).^10,17^ Serum SARS-CoV-2 antibodies were used as complementary diagnostic tests and were analyzed using commercial assays (Architect, Abbott Laboratories, and iFlash 1800, YHLO). SARS-CoV-2–specific IgG was not analyzed in CSF.

Lumbar puncture and venipuncture for CSF and blood sampling were performed in direct sequence. Clinical and neurologic status was recorded by the attending physician (infectious disease specialist or neurologist) in an electronic medical records database. Participant ethnicity was investigator observed using electronic medical records. The patient cohort was divided into groups according to neurologic status: neuroasymptomatic or neurosymptomatic. Disease severity was evaluated according to the World Health Organization Clinical Progression Scale.^18^

Two separate control groups were included in the study. At the time of antigen and cytokine analysis, prospective control samples were unavailable. Consequently, we used archived (−80 °C) CSF and serum samples from age-matched COVID-19–negative patient controls who had undergone a diagnostic lumbar puncture because of initial suspicion of CNS infection, with CSF analyses indicating no sign of ongoing CNS disease. For the other included CSF analyses, additional archived samples were unavailable. Instead, a second control group of healthy COVID-19–negative volunteers recruited prospectively during the pandemic was used.

### SARS-CoV-2 Antigen Detection

Detection of SARS-CoV-2 nucleocapsid and spike proteins was performed using MSD SPLEX CoV-2 N and MSD S-PLEX CoV-2 S assay kits (Meso Scale Discovery). The assays were run according to protocols in the kit package inserts (eMethods in the Supplement). The CSF and plasma samples were run undiluted (25 μL per well). Sample quantitation was achieved using a calibration curve generated using a recombinant antigen standard. For graphing and analysis, any concentrations below the limit of detection (LOD) were assigned the LOD value, and any concentrations above the highest calibration standard were assigned its value. The LOD values were 0.16 pg/mL for the nucleocapsid assay and 0.28 pg/mL for the spike assay, and the assay cutoffs (concentrations used for classifying samples as antigen positive) were 0.32 pg/mL for the nucleocapsid assay and 0.41 pg/mL for the spike assay (eMethods in the Supplement).

### Biomarker Analyses

IgG and albumin concentrations were measured by immunoturbidimetry on a Cobas instrument (Roche Diagnostics). The IgG index and albumin ratio were calculated as previously described.^19^ Cerebrospinal fluid neopterin was measured using a commercially available immunoassay (Brahms).^20^ Cerebrospinal fluid β_2_-microglobulin (β_2_M) was measured using the N latex β_2_M kit on the Atellica NEPH 630 System (Siemens Healthcare GmbH). The CSF NfL was measured using a previously described in-house sandwich enzyme-linked immunosorbent assay.^21^ The NfL concentrations were age adjusted and reported as the equivalent for 65 years of age as previously described.^22^ The CSF glial fibrillary acidic protein concentration was measured using an in-house enzyme-linked immunosorbent assay as previously described.^23^ Cytokine concentrations were measured using the novel S-PLEX Proinflammatory Panel I. The S-PLEX format provides signal enhancement that achieves sensitivity in femtograms per milliliters (eMethods in the Supplement). The CSF and plasma samples were tested undiluted.

### Statistical Analysis

All statistical analyses were performed using Prism software, version 9.0.0 (GraphPad Software) or SPSS, version 26 (IBM Inc). Numerical variables were log_10_ transformed where appropriate. Differences between patients with COVID and controls and differences in CSF NfL concentrations between the groups of patients with COVID-19 were estimated by Welch 2-sided *t* tests. Comparisons of other CSF biomarkers, as well as the first principal component of the cytokines neopterin and β_2_M, between the groups of patients with COVID-19 were performed using analysis of covariance, adjusting for time from symptom onset to CSF sampling. Because CSF NfL is known to increase gradually during the weeks after a CNS injury, this analysis was not adjusted for sampling time to avoid overadjustment. Correlations among CSF biomarkers were analyzed using partial correlation, adjusting for time of CSF sampling. Partial correlation coefficients are reported as *r* unless otherwise stated. Differences in baseline characteristics between patient groups were analyzed using *t* tests for continuous variables and Fisher exact test for categorical variables. All tests were 2-sided, and *P* < .05 was considered statistically significant.

## Results

### Study Participants

Forty-four patients (median [IQR] age, 57 [48-69] years; 30 male [68%] and 14 female [32%]), 10 healthy controls (median [IQR] age, 58 [54-60] years; 5 male [50%] and 5 female [50%]), and 41 patient controls (COVID negative without evidence of CNS infection) (median [IQR] age, 59 [49-70] years; 19 male [46%] and 22 female [54%]) were included in the study. All patients were admitted to the hospital with moderate (n = 26) or severe (n = 18) COVID-19 according to the World Health Organization Clinical Progression Scale.^18^ Twenty-one patients with COVID-19 were neuroasymptomatic, and 23 were neurosymptomatic. The neurosymptomatic group included patients with encephalopathy (n = 21), encephalitis (n = 1), and Guillain-Barré syndrome (n = 1). Encephalitis was defined as altered mental status and CSF pleocytosis (white blood cell count, ≥5/μL [to convert to ×10^9^/L, multiply by 0.001]), viral detection in CSF, or supporting evidence of CNS infection (neuroimaging or electroencephalography). Baseline characteristics in the different groups are shown in Table 1. The median (IQR) ages were 57 (48-69) years for the patients with COVID-19, 58 (54-60) years for the healthy controls, and 59 (49-70) years for the patient controls.

**Table 1.** Baseline Characteristics for the Patients With COVID-19 and Control Groups

Characteristic	Neuroasymptomatic patients (n = 21)	Neurosymptomatic patients (n = 23)	All patients (N = 44)	Healthy controls (n = 10)	Patient controls (n = 41)	*P* value (neurosymptomatic vs neuroasymptomatic)
Age, median (IQR), y	54 (49-60)	61 (45-75)	57 (48-69)	58 (54-60)	59 (49-70)	.13
Sex, male/female, No. (%)	14/7 (67/33)	16/7 (70/30)	30/14 (68/32)	5/5 (50/50)	19/22 (46/54)	>.99
Ethnicity						
European	20 (95)	22 (96)	42 (95)	NA	NA	NA
Middle Eastern	1 (5)	1 (4)	2 (5)	NA	NA	
WHO moderate severity, No. (%)	10 (48)	16 (70)	26 (59)	NA	NA	.22
WBC count, median (range), /μL	0 (0-7)	0 (0-261)	0 (0-261)	0	0	.28
Maximum CRP, median (range), U	150 (96-185)	110 (52-215)	135 (85-190)	NA	NA	.41
Minimum lymphocyte count, median (range), U	0.8 (0.6-1.0)	0.7 (0.6-1.2)	0.8 (0.6-1.1)	NA	NA	.51
Infection day of CSF sampling, median (range)	13 (3-21)	9 (3-16)	12 (3-21)	NA	NA	.008
Disease, No. (%)						
Hypertension	5 (24)	10 (44)	15 (34)	NA	NA	.51
Diabetes	2 (10)	3 (13)	5 (11)	NA	NA	>.99
Cardiovascular disease	1 (5)	2 (9)	3 (7)	NA	NA	>.99
Kidney disease	1 (5)	1 (4)	2 (4)	NA	NA	>.99
Medication, No. (%)						
Betamethasone	10 (48)	5 (22)	15 (11)	NA	NA	.02
Remdesivir	1 (5)	3 (13)	4 (9)	NA	NA	.43
Tocilizumab	2 (10)	NA	2 (4)	NA	NA	.17
VTE prophylaxis or anticoagulants, No. (%)	12 (57)	11 (48)	23 (52)	MA	NA	.18

Abbreviations: CRP, C-reactive protein; CSF, cerebrospinal fluid; NA, not applicable; VTE, venous thromboembolism; WBC, white blood cell; WHO, World Health Organization.

SI conversion factors: To convert CRP to milligrams per liter, multiply by 10; WBCs to ×10^9^/L, multiply by 0.001.

Neurosymptomatic patients tended to be older, had a lower proportion of severe COVID-19, and were consequently less likely to be treated with betamethasone (22% vs 48%; *P* = .02). The time from any COVID-19 symptom onset to CSF sampling was significantly shorter compared with the neuroasymptomatic group (median [range], 9 [3-16] vs 13 [3-21] days; *P* = .008). The CSF samples were acellular (white blood cell count, ≤4/μL) in all patients except in 2 neuroasymptomatic patients (white blood cell count, 6 and 7/μL) and 1 patient with encephalitis (white blood cell count, 261/μL). Three patients in the neurosymptomatic group died during hospitalization. Neuroimaging was performed in a subset of participants (eResults in the Supplement).

### Viral Detection

**Figure 1.**
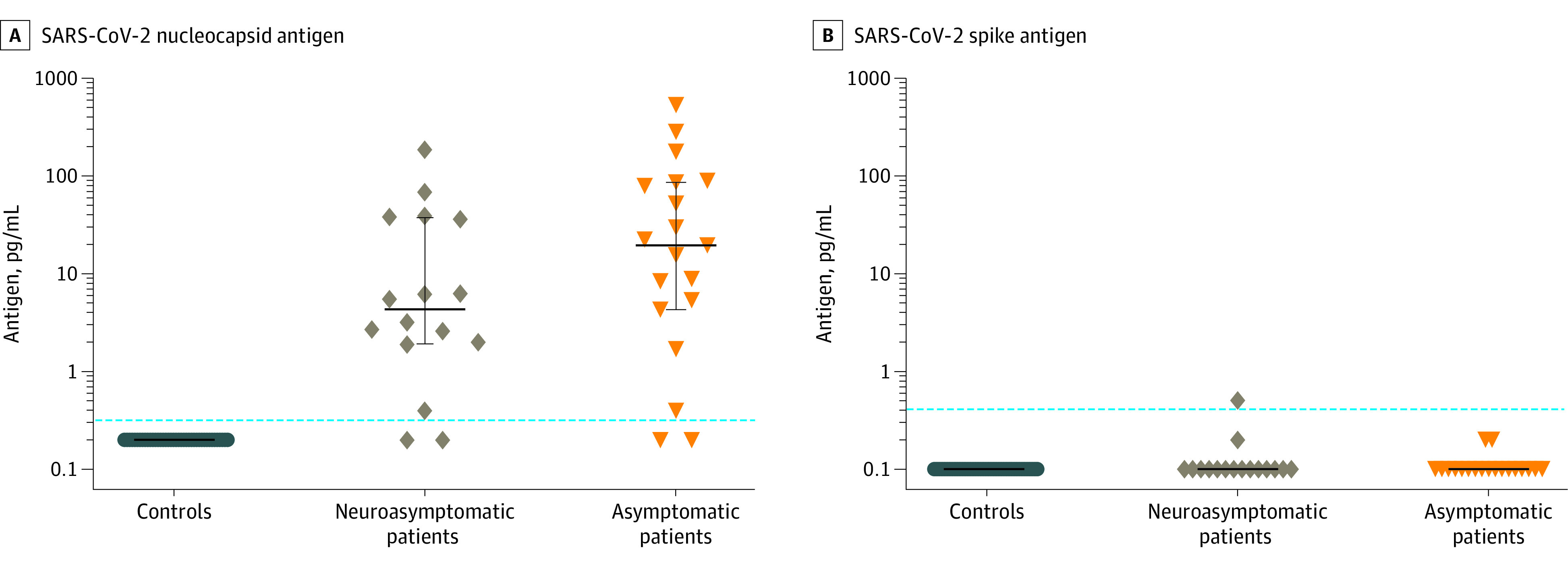
SARS-CoV-2 Cerebrospinal Fluid (CSF) Antigen Detection Detection of nucleocapsid antigen and spike antigen in CSF in patient groups and controls. A, Nucleocapsid antigen was positive in 31 of 35 (14 of 16 neuroasymptomatic and 17 of 19 neurosymptomatic) patients and 0 of 41 controls. B, Spike antigen was positive in 1 patient (0 of 41 controls). Positive assay cutoffs (dashed lines) were 0.32 pg/mL for nucleocapsid antigen and 0.41 pg/mL for spike antigen. Points represent individual values; lines and whiskers represent medians and IQRs.

All CSF samples tested negative for SARS-CoV-2 RNA, whereas 14 patients (11 of 20 neuroasymptomatic and 3 of 15 neurosymptomatic) had detectable viral RNA in plasma. The median cycle threshold (Ct) value for detectable plasma samples was 36.0 (range, 31.3-38.0). Concentrations of serum and CSF SARS-CoV-2 nucleocapsid and spike antigens are given in Table 2. The SARS-CoV-2 N-Ag was detectable in CSF in 31 of 35 patients (89%) (0 of 41 controls). The measured CSF N-Ag concentrations were not significantly different between the groups of patients with COVID-19 according to neurosymptoms (Figure 1) or disease severity (eTable 1 in the Supplement) after adjustment for CSF sampling day. Cerebrospinal fluid SARS-CoV2 spike antigen (S-Ag) was positive in 1 patient. Serum-CSF ratios are given in the eResults in the Supplement.

**Table 2.** Biomarker Concentrations^a^

Biomarker	Neuroasymptomatic patients (n = 21)	Neurosymptomatic patients (n = 23)	Healthy controls (n = 10)	Patient controls (n = 41)	*P* value (patients vs controls)	Neurosymptomatic patients vs neuroasymptomatic patients	
						Ratio (95% CI)^b^	*P* value
**SARS-CoV-2 antigen**							
CSF N-Ag positive, No./No.	14/16	17/19	NA	0/41	<.001	1.2 (0.1-19)^c^	>.99
N-Ag CSF, pg/mL	4.4 (1.9-37.7)	19.7 (4.3-86.4)	NA	Not detected	NA	1.5 (0.3-7.0)	.63
N-Ag serum, pg/mL	80.8 (7.5-2482.2)	725.9 (100-14 313.5)	NA	Not detected	NA	2.0 (0.5-7.7)	.28
CSF S-Ag positive, No./No.	1/16	0/19	NA	0/41	>.99	∞ (0.03-∞)^c^	>.99
S-Ag CSF, median (range), pg/mL	0 (0-0.5)	0 (0-0.2)	NA	Not detected	NA	0.8 (0.7-1.1)	.17
S-Ag serum, pg/mL	0.5 (0-51.3)	7 (0.9-216.1)	NA	Not detected	NA	1.1 (0.2-7.0)	.89
**CSF biomarker concentrations**							
WBC count ≥5/μL	2	1	0	NA	.24	0.4 (0.01-9.1)^c^	.60
Albumin ratio	6.1 (4.6-7.4)	6.5 (4.2-9.8)	5.3 (4.4-6.5)	NA	.74	1.3 (0.8-2.0)	.34
IgG index	0.39 (0.36-0.44)	0.41 (0.38-0.43)	0.43 (0.41-0.46)	NA	.03	1.0 (0.9-1.1)	.48
Neopterin, nmol/L	21.7 (17.3-43.7)	47.1 (33.6-65.4)	4.9 (4.8-6.3)	NA	<.001	1.4 (0.99-1.9)	.06
β_2_M, mg/L	2.1 (1.5-3.0)	2.7 (1.9-3.3)	1.1 (1.0-1.2)	NA	<.001	1.0 (0.8-1.3)	.81
NfL (age-adjusted), ng/L	960 (654-1114)	974 (734-2038)	618 (489-786)	NA	.002	1.5 (0.9-2.5)	.12
GFAp, ng/L	220 (160-360)	335 (230-717)	240 (178-363)	NA	.57	1.3 (0.8-2.2)	.28
**CSF cytokines, fg/mL**							
IL-6	5475 (3382-11 971)	15 614 (9703-47 791)	NA	3598 (2445-5434)	<.001	1.8 (0.97-3.5)	.06
IL-10	132 (95-188)	275 (202-491)	NA	112 (74-150)	<.001	1.3 (0.8-1.9)	.24
TNF-α	25 (21-32)	46 (33-52)	NA	18 (15-25)	<.001	1.4 (0.99-1.8)	.06
IL-2	43 (28-93)	82 (45-129)	NA	42 (28-65)	.02	1.3 (0.9-2.1)	.19
IFN-γ	21 (17-81)	86 (47-172)	NA	33 (26-67)	.22	2.0 (1.1-3.7)	.03
IL-12p70	46 (0-64)	37 (0-66)	NA	0 (0-58)	.56	0.9 (0.7-1.2)	.66
IL-4	0 (0-0)	0 (0-0)	NA	0 (0-0)	.31	1.0 (0.9-1.1)	.69
IL-1β	22 (0-30)	29 (17-33)	NA	0 (0-24)	.12	1.2 (0.7-2.0)	.56
IL-17A	46 (33-55)	35 (28-58)	NA	40 (29-58)	.74	0.9 (0.6-1.2)	.48

Abbreviations: β_2_M, β_2_-microglobulin; CSF, cerebrospinal fluid; GFAp, glial fibrillary acidic protein; IFN, interferon; IL, interleukin; NA, not applicable; N-Ag, nucleocapsid antigen; NfL, neurofilament light protein (reported as the equivalent for 65 years of age); S-Ag, spike antigen; TNF, tumor necrosis factor; WBC, white blood cell.

SI conversion factor: To convert WBCs to ×10^9^/L, multiply by 0.001.

^a^ All CSF and serum biomarker concentrations are reported as unadjusted assay results and are listed as median (IQR) unless otherwise indicated. All statistical comparisons of biomarker concentrations between the groups of patients with COVID-19 except NfL were adjusted for time from symptom onset to CSF sampling.

^b^ Ratio between geometric means unless otherwise indicated.

^c^ Odds ratio.

### CSF Biomarkers

**Figure 2.**
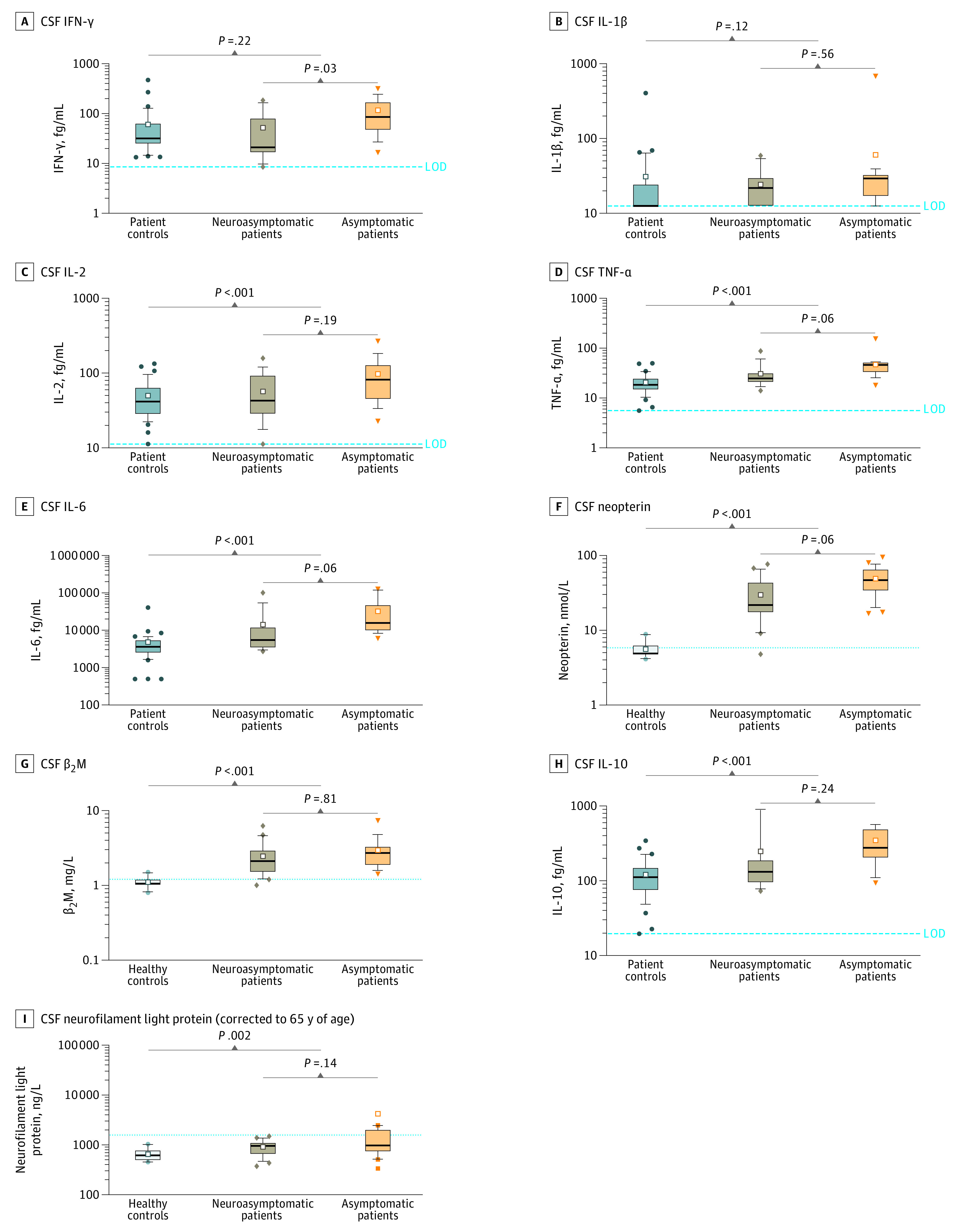
Neuroinflammatory Cerebrospinal Fluid (CSF) Biomarker Profiles in Groups of Patients With COVID-19 and Controls Profiles of CSF biomarkers of inflammation/immune activation (A-H) and neuroaxonal injury (I) in the 21 neuroasymptomatic and 23 neurosymptomatic patient groups, patient control groups (n = 41), and healthy control groups (n = 10). Boxes indicate the median and IQR, whiskers indicate 10th and 90th percentiles, small white squares indicate means, and values above or below are drawn as individual points. Concentrations are shown as unadjusted values. Dashed lines indicate assay levels of detection (LODs). Dotted lines indicate upper normal reference values. β_2_M indicates β_2_-microglobulin; IFN-γ, interferon γ; IL, interleukin; TNF-α, tumor necrosis factor α.

Cerebrospinal fluid biomarker concentrations are given in Table 2. The albumin ratio was above the upper normal reference (10.2) in 5 of 43 patients (12%), but no significant difference was seen in the albumin ratio between the groups of patients with COVID-19 and the control groups. All patients with COVID-19 and controls had a normal IgG index. Patients with COVID-19 had significantly higher CSF concentrations of neopterin, β_2_M, interleukin (IL) 2, IL-6, IL-10, and tumor necrosis factor α (TNF-α) compared with controls (Table 2 and Figure 2).

Comparing the groups of patients with COVID-19, significantly higher concentrations of several biomarkers were seen in neurosymptomatic compared with neuroasymptomatic patients (Figure 2). However, because the CSF biomarker concentrations had similar trajectories depending on when the CSF sample was drawn, we adjusted the analysis for time from COVID-19 symptom onset to CSF sampling to avoid overestimation of the effect size because time of sampling was a significant covariate for CSF biomarker concentrations. Details of CSF biomarkers on the basis of sampling time are shown in eFigure 1 in the Supplement. Because median time from symptom onset to CSF sampling was significantly shorter in neurosymptomatic patients, we included sampling day in the model. After adjusting for CSF sampling day, the higher observed concentrations of IFN-γ, neopterin, IL-6, and TNF-α in the neurosymptomatic group (Figure 2) remained statistically significant for IFN-γ (neurosymptomatic-neuroasymptomatic ratio, 2.0; 95% CI, 1.1-3.7; *P* = .03), whereas CSF neopterin (ratio, 1.4; 95% CI, 0.99-1.9; *P* = .06), IL-6 (ratio, 1.8; 95% CI, 0.97-3.5; *P* = .06), and TNF-α (ratio, 1.4; 95% CI, 0.99-1.8; *P* = .06) fell just above the threshold for significance (Table 2). We compared the first principal component of the cytokines, β_2_M, and neopterin between the groups of patients with COVID-19 as a single test of difference in immunomarkers and found a significant difference (0.98; 95% CI, 0.36-1,61; *P* = .003), which remained significant after adjustment for time of sampling (0.54; 95% CI, 0.03-1.05; *P* = .04).

Age-adjusted median (IQR) CSF NfL concentrations were significantly higher in patients with COVID-19 compared with controls (960 [673-1307] ng/L vs 618 [489-786] ng/L; *P* = .002), whereas no significant difference was found between the groups of patients with COVID-19 (Table 2 and Figure 2). Notably, 7 of 20 neurosymptomatic patients (35%) had concentrations of CSF NfL equivalent for 65 years of age above the upper normal reference (1577 ng/L), whereas CSF NfL equivalent for 65 years of age concentrations were within normal limits in all individuals in the other groups. We found no significant differences in any of the CSF biomarkers in patients with moderate compared with severe disease after adjusting for CSF sampling day.

**Figure 3.**
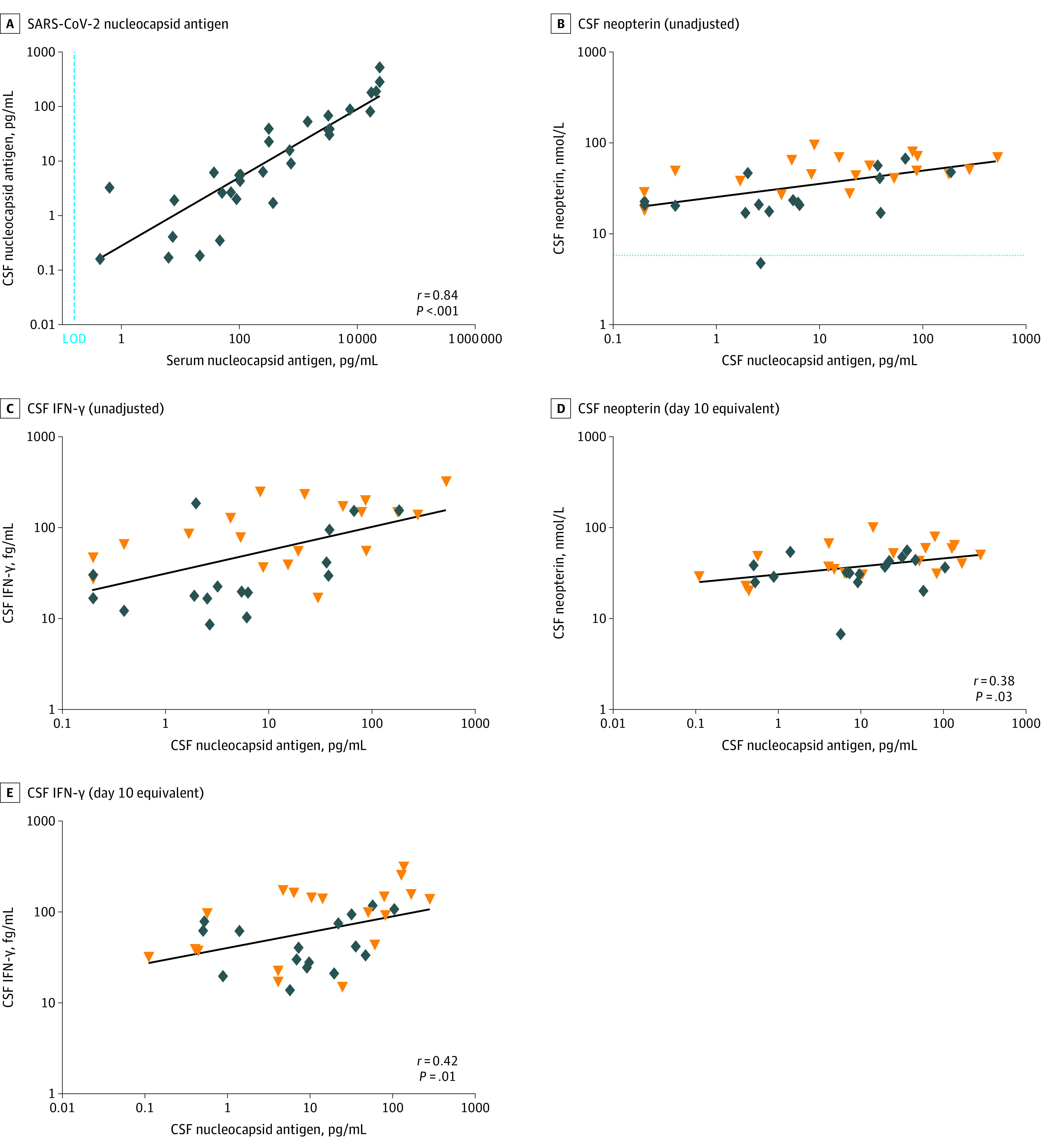
SARS-CoV-2 Nucleocapsid Antigen Correlations A, A close correlation was found between cerebrospinal fluid (CSF) and serum SARS-CoV-2 nucleocapsid antigen. Individual points are shown using unadjusted nucleocapsid antigen concentrations in patients with COVID-19. The median (IQR) ratio of serum-CSF nucleocapsid antigen was 42 (range, 18-89). B and C, CSF neopterin and interferon (IFN) γ concentrations are shown as unadjusted individual values in relation to CSF nucleocapsid antigen. B, Dotted line represents upper normal reference for CSF neopterin. D and E, CSF SARS-CoV-2 nucleocapsid antigen was significantly correlated with CSF neopterin (D) and CSF IFN-γ (E) adjusted for time of CSF sampling. Individual values are adjusted for time of sampling and are reported as the day 10 equivalent concentration. All correlation coefficients (*r*) and statistical significances were estimated using partial correlation adjusted for time from symptom onset to CSF sampling day.

A close correlation was found between serum and CSF concentrations of SARS-CoV-2 N-Ag (*r* = 0.84; *P* < .001). Initial analysis also indicated a significant association between CSF N-Ag and the immune activation markers CSF neopterin, β_2_M, and IFN-γ, but after adjusting for CSF sampling day, correlations remained significant only for CSF neopterin (*r* = 0.38, *P* = .03) and IFN-γ (*r* = 0.42; *P* = .01) (Figure 3).

### Serum Biomarkers

Serum biomarker concentrations followed a similar pattern of immune activation compared with CSF. Additional statistical differences were seen for several biomarkers between the groups of patients with COVID-19 in serum compared with CSF, as well as between patients with COVID-19 and controls. Time-adjusted differences in serum neopterin, β_2_M, TNF-α, IFN-γ, IL-4, and IL-2 were seen between the groups of patients with COVID-19, with higher concentrations found in neurosymptomatic patients. Compared with controls, patients with COVID-19 had higher serum concentrations of all biomarkers except IL-12p70 and IL-1β (eTable 2 and eFigure 2 in the Supplement).

We used an albumin ratio–adjusted biomarker index ([CSF concentration/serum concentration]/albumin ratio) to explore differences in intrathecal biomarker proportions between the patient groups. Of all studied biomarkers, only IL-4 showed a significant difference (neurosymptomatic-neuroasymptomatic ratio, 2.7; 95% CI, 1.1-6.7; *P* = .03), suggesting that CNS symptoms were not associated with selective intrathecal production of the inflammatory biomarkers.

## Discussion

In this study of patients hospitalized with acute COVID-19, we found that despite the absence of viral RNA, SARS-CoV-2 N-Ag was readily detectable in CSF in most individuals using a novel ultrasensitive assay. Furthermore, CSF N-Ag was correlated with the CSF immune activation biomarkers neopterin and IFN-γ. Our findings indicate that SARS-CoV-2 viral antigens are associated with a CNS immune response without apparent viral invasion of the CNS. Although CSF N-Ag concentrations were not significantly different between patient groups, patients with COVID-19 had higher concentrations of CSF NfL compared with controls, and neurosymptomatic patients had a more marked immune activation biomarker profile, suggesting that the magnitude of the CNS immune response, possibly triggered by viral components, contributes to the neuropathogenesis of COVID-19.

Although the neuroinvasive capacity of SARS-CoV-2 is questionable,^6,7,8,9,24,25,26,27,28^ intrathecal immune activation is a prominent feature in several CSF studies.^4,10,11,29^ The presence of SARS-CoV-2 N-Ag may partially explain the apparent discrepancy between the lack of viral RNA detection and the CNS immune response in COVID-19, but antigen detection has rarely been included in CSF studies. Nucleocapsid or spike antigen was not detected in any CSF samples of 13 patients with cancer and severe COVID-19 with neurologic symptoms using a commercial enzyme-linked immunosorbent assay, presumably related to assay sensitivity, although the time of sampling may have contributed to the lack of antigen detection.^29^ In plasma, as confirmed in the current study, N-Ag was detected in most patients during early SARS-CoV-2 infection.^15^ We found a high degree of correlation between serum and CSF N-Ag concentrations (*r* = 0.84; *P* < .001) and a median serum-CSF ratio of 42 (range, 18-89), suggesting that CSF N-Ag originates from systemic sources, either by passive diffusion across the blood-brain barrier or as a consequence of viral interaction with the neurovascular unit, possibly including infection of neurovascular pericytes.^30,31,32^ The markedly lower serum concentrations in combination with the potential loss of S-Ag by binding to angiotensin-converting enzyme 2 on cells lining the ventricular system may have influenced the low detection rate of S-Ag in CSF.

Patients with COVID-19 had significantly increased CSF concentrations of neopterin, β_2_M, IL-2, IL-6, IL-10, and TNF-α compared with controls, indicating CNS-specific inflammation and confirming previous findings of increased neopterin and β_2_M in patients with COVID-19 with neurologic symptoms.^10,11^ Although increased CSF concentrations of IL-6 and TNF-α have also been described previously,^11,12,33^ results are less consistent across studies.^13,34^ Although other CNS infections and inflammatory conditions can generate significantly higher inflammatory CSF biomarker concentrations,^13,35^ the CSF inflammatory biomarker profile seen in patients with COVID-19 in our study remains a consistent finding and adds to previous reports^11,29,36,37^ indicating the importance of CNS immune activation in the neuropathogenesis of COVID-19.

The underlying mechanism of neurologic symptoms (particularly encephalopathy) during COVID-19 is not clear. Of interest, we found that patients with neurologic symptoms had significantly higher time-adjusted CSF concentrations of IFN-γ and a more marked inflammatory biomarker profile compared with neuroasymptomatic patients. Age-adjusted CSF NfL, representing neuroaxonal injury, was also significantly higher in patients with COVID-19 compared with controls. In addition, all patients with CSF NfL concentrations above the upper normal reference were found in the neurosymptomatic group. Overall, we found a notable correlation between CSF NfL and CSF TNF-α, IL-1β, and IL-17A, suggesting that immune cell activation and the CNS inflammatory response may result in neuroaxonal injury. Altogether, these findings indicate that the CNS immune response may be an important driver of CNS pathogenesis, similar to other CNS inflammatory conditions or infections.^11,13,29,38^ The principal difference between patient groups appeared to be in the magnitude of the immune response, suggesting that neuropathogenesis in COVID-19 with neurologic symptoms represents a continuum of inflammation, where the intensity of the inflammatory response (likely in combination with individual premorbid vulnerability) may be a determining factor in the development of CNS symptoms. In turn, CNS symptoms may reflect harmful effects on the brain measurable as neuroaxonal injury (CSF NfL). Notably, CSF biomarkers followed a similar pattern compared with serum, where differences in biomarkers between groups of patients with COVID-19 as well as controls were more evident, indicating that the CNS inflammatory response likely constitutes a continuous part of the systemic infection. However, qualitative differences in the immune response in neurosymptomatic compared with neuroasymptomatic patients remains a possibility that needs to be investigated further. As described by others,^14^ no significant differences were found in CSF glial fibrillary acidic protein concentrations between any of the patient groups and controls, indicating that astrocyte activation may not be a major contributor to CNS pathogenesis in COVID-19.

Few previous reports have included CSF analyses from patients with COVID-19 without neurologic symptoms. In a small study^33^ of patients in the intensive care unit, neurologic syndromes were associated with higher CSF concentrations of several cytokines and chemokines. However, observations made exclusively in patients in the intensive care unit may be subject to bias that can be attributed to critical illness and organ failure. We did not find any significant differences in CSF biomarkers in patients with moderate compared with severe COVID-19. Moreover, the lower proportion of patients with severe COVID-19 in those with neurologic symptoms compared with the neuroasymptomatic group (30% vs 52%) further suggests that neurologic manifestations represent a specific pathological process within the CNS that cannot be attributed solely to systemic infection.

The time from symptom onset to CSF sampling was an important covariate for CSF biomarker concentrations. The timing of CSF sampling has varied considerably in previous studies.^11,12,13,14,39^ Given the inflammatory character of acute COVID-19, sampling at a later time may not reflect the processes that initiate CNS pathology. Moreover, few studies have adjusted for CSF sampling day, which may have influenced results.^13^ In the current study, all CSF samples were collected during the acute phase of COVID-19 (median of 12 days from symptom onset). Although we believe this timing is important for evaluating viral antigen detection and initial CNS immune responses, the CSF NfL is known to have a slower initial kinetic and longer half-life, where additional CSF sampling later in the disease course would be of interest for further evaluating the association of CNS inflammation with markers of neuronal injury.^40,41^

### Strengths and Limitations

Strengths of this study include the prospective design, the inclusion of neuroasymptomatic as well as neurosymptomatic patients, and the inclusion of control groups. However, the study also has several limitations. The sample size, although large in comparison with other published reports,^11,12,42^ is still relatively small, and neuroimaging was not included consistently. Encephalopathy was the dominating neurologic manifestation, although the definition of encephalitis differed slightly from those used in some previous studies^43^ of COVID-19 with neurologic symptoms. Furthermore, the study did not include nonhospitalized patients with mild COVID-19. The small study size and exploratory approach prohibited evaluation of the potential prognostic value of the studied biomarkers. Of necessity, the study included 2 different control groups. Notably, the patient controls used for antigen and cytokine analyses had CSF samples drawn for clinical suspicion of CNS infection. Although no infection was subsequently diagnosed, some individuals may have had other undiagnosed CNS conditions that potentially affected CSF biomarker concentrations. It is highly unlikely that this would exaggerate differences compared with the COVID-19 samples, although we cannot rule out an opposite effect. In addition, we cannot exclude that other residual confounding factors may have influenced results.

## Conclusions

In this cross-sectional study of Swedish adults with COVID-19 infection and neurologic symptoms, compared with control participants, SARS-CoV-2 N-Ag was detectable in CSF in most patients with COVID-19, despite the absence of detectable CSF viral RNA, and was correlated with CNS immune activation. Furthermore, neurosymptomatic patients had a more pronounced immune activation profile compared with neuroasymptomatic patients, and patients with COVID-19 had signs of neuroaxonal injury compared with controls. These observations could not be attributed to differences in COVID-19 severity, where no differences in CSF biomarkers were seen in patients with moderate compared with severe disease.

Although confirmatory studies evaluating the significance of viral antigen detection in CSF are needed, these results suggest that viral components may contribute to CNS immune responses without direct viral invasion into the CSF compartment, which could partly explain the apparent discrepancy between the scarcity of CSF viral RNA detection and the marked CNS inflammatory response seen in patients with COVID-19 with neurologic symptoms. The results of this study also highlight the importance of neurologic symptoms, including encephalopathy. These findings have important potential implications for clinical treatment of patients with COVID-19, including the use of antiviral therapies, as well as for the continued importance of including CSF analyses in future studies of CNS pathogenesis and treatment strategies.

## Notes

Supplement. **eMethods.** Supplementary Methods **eResults.** Supplementary Results **eTable 1.** CSF Biomarker Concentrations in Moderate and Severe COVID-19 **eTable 2.** Serum Biomarker Concentrations in COVID Patient and Control Groups **eFigure 1.** CSF Biomarker Concentrations in Relation to Sampling Time **eFigure 2.** Inflammatory Serum Biomarker Profiles in COVID-19 Patient Groups and Controls
eReferences
