# Error in Figures 1 and 2

**DOI:** 10.1001/jamanetworkopen.2022.21406

**Published:** 2022-06-22

In the Original Investigation titled “Viral Antigen and Inflammatory Biomarkers in Cerebrospinal Fluid in Patients With COVID-19 Infection and Neurologic Symptoms Compared With Control Participants Without Infection or Neurologic Symptoms,”^1^ published May 23, 2022, in Figures 1 and 2, the label “Neurosymptomatic patients” had been incorrectly written as “Asymptomatic patients.” This article has been corrected.^1^
