# Supplementary material for: Viral Antigen and Inflammatory Biomarkers in Cerebrospinal Fluid in Patients With COVID-19 Infection and Neurologic Symptoms Compared With Control Participants Without Infection or Neurologic Symptoms
Source: JAMA Netw Open. 2022 May 23;5(5):e2213253. doi: 10.1001/jamanetworkopen.2022.13253 (PMC9127556; doi:10.1001/jamanetworkopen.2022.13253)
Supplement: Supplement. — eMethods. Supplementary Methods eResults. Supplementary Results eTable 1. CSF Biomarker Concentrations in Moderate and Severe COVID-19 eTable 2. Serum Biomarker Concentrations in COVID Patient and Control Groups eFigure 1. CSF Biomarker Concentrations in Relation to Sampling Time eFigure 2. Inflammatory Serum Biomarker Profiles in COVID-19 Patient Groups and Controls eReferences [file jamanetwopen-e2213253-s001.pdf]

Supplementary Online Content

Edén A, Grahn A, Bremell D, et al. Viral antigen and inflammatory biomarkers in cerebrospinal fluid in patients with COVID-19 infection and neurologic symptoms compared with control participants without infection or neurologic symptoms. *JAMA Netw Open*. 2022;5(5):e2213253. doi:10.1001/jamanetworkopen.2022.13253

eMethods. Supplementary Methods

eResults. Supplementary Results

eTable 1. CSF Biomarker Concentrations in Moderate and Severe COVID-19

eTable 2. Serum Biomarker Concentrations in COVID Patient and Control Groups

eFigure 1. CSF Biomarker Concentrations in Relation to Sampling Time

eFigure 2. Inflammatory Serum Biomarker Profiles in COVID-19 Patient Groups and Controls

eReferences

This supplementary material has been provided by the authors to give readers additional information about their work.

© 2022 Edén A et al. *JAMA Network Open.*

eMethods. Supplementary Methods

Viral diagnostic methods

Nucleic acid from 200 µL nasal swab medium, plasma or CSF was extracted from the clinical samples in a MagNA Pure 96 instrument using the Total Nucleic Acid isolation kit (Roche, Basel, Switzerland). RT-PCR targeting the RNA-dependent RNA polymerase (RdRP) region was performed by a QuantStudio 6 instrument (Applied Biosystems, Foster City, CA, USA) using a probe and the primers RdRP_Fi, GTCATGTGTGGCGGTTCACT and RdRP_Ri, CAACACTATTAGCATAAGCAGTTGT[1]. Viral load was estimated using the cycle threshold (Ct) values using formula $(47 - Ct)/3.4 = \log10$ copies/sample. Ct values ≤38 were considered as positive.

MSD S-PLEX CoV-2 N, MSD S-PLEX CoV-2 S, and MSD S-PLEX Proinflammatory Panel I assay design

The MSD S-PLEX assay kits (Meso Scale Discovery, Rockville, MD) employ a sandwich immunoassay format and electrochemiluminescence (ECL) detection. The assays are carried out in specially designed 96-well plate consumables having integrated screen-printed carbon ink electrodes on the bottom of each well that are used as solid-phase supports for binding reactions, and as the source of electrical energy for inducing ECL from ECL labels in binding complexes on their surfaces. The kits use MSD’s ultra-sensitive S-PLEX ECL format, which provides additional signal enhancement and sensitivity relative to conventional ECL formats. Sample quantitation was achieved using a calibration curve generated with a recombinant antigen standard, and fit to a four parameter logistic (4PL) model. The LOD for all S-PLEX assays was determined as the concentration (based on a 4PL fit to a calibration curve) that provides a signal 2.5 standard deviations above the blank signal. For graphing and analysis, any concentrations below the limit of detection (LOD) were assigned the LOD value, and any concentrations above the highest calibration standard were assigned the concentration of the highest calibration standard.

The N antigen assay[2-4] utilizes recombinant full-length N-protein as a standard, and monoclonal capture/detection antibodies generated by MSD against the full-length recombinant N protein. The S antigen assay[3] utilizes recombinant receptor binding domain (RBD) of the S1 subunit of the spike protein as a standard, and monoclonal capture/detection antibodies generated by MSD against recombinant RBD. The assay cut-offs for classifying samples as N or S antigen negative or positive were established in previous clinical studies of respiratory or plasma samples[2,3].

All S-PLEX cytokine assays used recombinant cytokines as calibrator material and monoclonal antibodies as capture and detection antibodies. Anchoring of the calibrators to WHO or NIBSC standards is described in the package insert. LLOQ of the assays was defined as the lowest concentration that gave consistently CVs of less than 25% (<30% for IL-1b) and analyte recovery between 80% and 120% (75%-125% for IL-1b). LOD and LLOQ of the cytokines were as follows (all in fg/mL): IL-1b: 12, 170; IL-2: 11, 80; IL-4: 7, 27; IL-6: 5, 54; IL-10: 20, 170; IL-12(p70): 34, 490; IL-17A: 24, 130; IFNg: 9, 130; and TNFa 6, 53. Inserts: Meso Scale Discovery. Package Insert, S-PLEX SARS-CoV-2 N Kit. Accessed October 30, 2021. Meso Scale Discovery. Package Insert, S-PLEX SARS-CoV-2 Spike Kit. Accessed October 30, 2021.

© 2022 Edén A et al. *JAMA Network Open.*

eResults. Supplementary Results

Neuroimaging

Neuroimaging was not included in the study protocol, and availability of imaging was limited in the early part of the pandemic due to infection prevention and control measures. However, brain computed tomography (CT) scan was performed in 19/23, and MRI in 4/23 patients in the NS-group. No evidence of encephalitis, acute hemorrhage, microhemorrhage or ischemia was found in imaging studies, while signs of global cortical atrophy were found in one, and small vessel disease in 4 patients.

Serum to CSF antigen ratios

The median (IQR) ratio of serum:CSF N-Ag was 42 (18-89). Overall, concentrations of serum N-Ag were markedly higher compared with S-Ag concentrations (Table 2). Although the borderline concentrations and small number of detectable samples of S-Ag in CSF prohibited an accurate calculation of the corresponding ratio for serum:CSF S-Ag ratio, the number of CSF samples with positive S-Ag detection was within an expected range considering the measured serum concentrations of S-Ag given that S-Ag had a comparable serum:CSF ratio to N-Ag.

Additional CSF biomarker associations

Several additional time-adjusted associations were also seen between the individual CSF biomarkers. CSF neopterin was closely correlated to CSF β_2M (r=0,65; P=.001), and also to CSF IL-1β (r=0.40, P=.02) and IFN-γ (r=0.39, P=.02). CSF β2M was additionally correlated to the CSF cytokines IL-12p70 (r=0,44, P=.01), IL-2 (r=0,37; P=.04), IFN-γ (r=0.43, P=.01) and IL-17A (r=0.49, P=.004). Age-adjusted CSF NfL(65) was correlated to CSF concentrations of TNFα (r=0.68; P<.001), IL-2 (r=0.36; P=.04), IL-1β (r=0.67; P<0.001) and IL-17A (r=0.47; P=.006). Correlations were also seen between CSF GFAp and the albumin ratio (r=0.43, P=.02) as well as CSF TNFα (r=0.40; P=.05) and IFN-γ (r=0.61; P<0.01).

© 2022 Edén A et al. *JAMA Network Open.*

**eTable 1.** CSF Biomarker Concentrations in Moderate and Severe COVID-19		
**WHO clinical progression scale category**	**Moderate**	**Severe**
	**(n=26)**	**(n=18)**
**SARS-CoV-2 antigen**		
Detectable N-Ag, number	19/21	12/14
Median (IQR) N-Ag csf (pg/mL)	8.4 (1.8–38.6)	12.9 (2.5–179.4)
Median (IQR) N-Ag serum (pg/mL)	318.8 (88.2–3205.3)	51.3 (7.1–19138.4)
Detectable S-Ag	1	3
Median (range) S-Ag, csf (pg/mL)	0 (0–0.2)	0 (0–0.5)
Median (IQR) S-Ag, serum (pg/mL)	2.1 (0.6–14.2)	0.6 (0–311.7)
**CSF biomarker concentrations**		
WBC count (cells/µL), median (range)	0 (0–261)	0 (0–6)
Albumin ratio	6.3 (3.6–7.3)	6.7 (4.9–9.8)
IgG-index	0.4 (0.37–0.44)	0.41 (0.36–0.43)
Neopterin (nmol/L)	40.9 (21.9–58.8)	25.5 (19.9–51.7)
β2M (mg/L)	2.6 (1.9–3.6)	2.1 (1.5–3.2)
NfL (65) (ng/L)	918 (660–1464)	1025 (687–1114)
GFAp (ng/L)	260 (160–490)	260 (168–435)
**Cytokines (fg/mL)**		
IL-6	12793 (8185–37970)	7105 (3324–15567)
IL-10	228 (165–507)	141 (105–235)
TNFα	45 (25–52)	27 (24–36)
IL-2	82 (47–128)	37 (28–75)
IFNγ	77 (33–163)	27 (17–140)
IL-12p70	41 (34–65)	37 (34–69)
IL-4	0 (0–0)	0 (0–0)
IL-1b	24 (13–32)	28 (13–34)
IL-17A	47 (31–56)	35 (32–59)

CSF and serum biomarker concentrations in patients with moderate and severe COVID-19 according to the WHO clinical progression scale. Values are listed as median (IQR) unless otherwise stated. CSF, cerebrospinal fluid; SARS-CoV-2, severe acute respiratory syndrome coronavirus-2; N-Ag, nucleocapsid antigen; S-Ag, spike antigen; WBC, white blood cell; IgG, immunoglobulin G; β2M, β2-microglobulin; NfL (65), neurofilament light protein (age-adjusted); GFAp, glial fibrillary acidic protein; IL, interleukin; TNF, tumor necrosis factor; IFN, interferon.

© 2022 Edén A et al. *JAMA Network Open.*

eTable 2. Serum Biomarker Concentrations in COVID Patient and Control Groups

	**CNS -**	**CNS +**	**Healthy controls**	**Patient controls**	**Patients vs controls**	**CNS + vs CNS -**	
	**(n=21)**	**(n=23)**	**(n=10)**	**(n=41)**			
**Serum biomarker concentrations**					**P**	**Ratio[a] (CI)**	**P**
Neopterin (nmol/L)	20.3 (12.8-27.2)	43.1 (30.6–56.2)	6.1 (5.2–7.6)	–	**<.001**	**1.7 (1.2-2.4)**	**.003**
β2M (mg/L)	2.3 (2.0–2.5)	3.3 (2.8–4.5)	1.5 (1.3–1.6)	–	**<.001**	**1.5 (1.2-1.9)**	**.001**
IL-6 (fg/mL)	6393 (1866–36153)	62396 (16728–99246)	–	1715 (1188–2355)	**<.001**	3.8 (.96-11)	.06
IL-10 (fg/mL)	4911 (2319–6830)	11987 (2834–25458)	–	576 (435–926)	**<.001**	2.6 (.8-8.4)	.11
TNF-α (fg/mL)	1227 (1102–1480)	2312 (1542–3304)	–	613 (483–734)	**<.001**	**1.8 (1.2-2.7)**	**.007**
IL-2 (fg/mL)	317 (228–686)	1108 (354–2003)	–	122 (59–158)	**<001**	**2.4 (1.2-4.8)**	**.02**
IFN-γ (fg/mL)	1948 (499–4280)	21970 (1944–46028)	–	816 (560–1213)	**<.001**	**5.3 (1.5-18)**	**.01**
IL-12p70 (fg/mL)	167 (167–324)	563 (220–848)	–	273 (199–465)	.81	1.6 (.8-3.4)	.21
IL-4 (fg/mL)	25 (9–68)	78 (37–158)	–	97 (50–136)	**.04**	**2.4 (1.1-5.5)**	**.04**
IL-1β (fg/mL)	0 (0–278)	514 (0–1063)	–	0 (0–434)	.06	1.8 (.9-3.4)	.09
IL-17A (fg/mL)	396 (278–808)	720 (578–1459)	–	332 (195–672)	**.003**	1,7 (.96-3.0)	.07

Serum biomarker concentrations in COVID patient and control groups. All biomarker concentrations are reported as unadjusted assay results, and are listed as median (IQR) unless otherwise stated. All statistical comparisons of biomarker concentrations between COVID patient groups were adjusted for time from symptom onset to CSF sampling.
[a]Ratio (CNS+/CNS-) between geometric means; SARS-CoV-2, severe acute respiratory syndrome coronavirus-2; β2M, β2-microglobulin; IL, interleukin; TNF, tumor necrosis factor; IFN, interferon; CNS -, neuroasymptomatic; CNS +, neurosymptomatic.

eFigure 1. CSF Biomarker Concentrations in Relation to Sampling Time

© 2022 Edén A et al. *JAMA Network Open.*

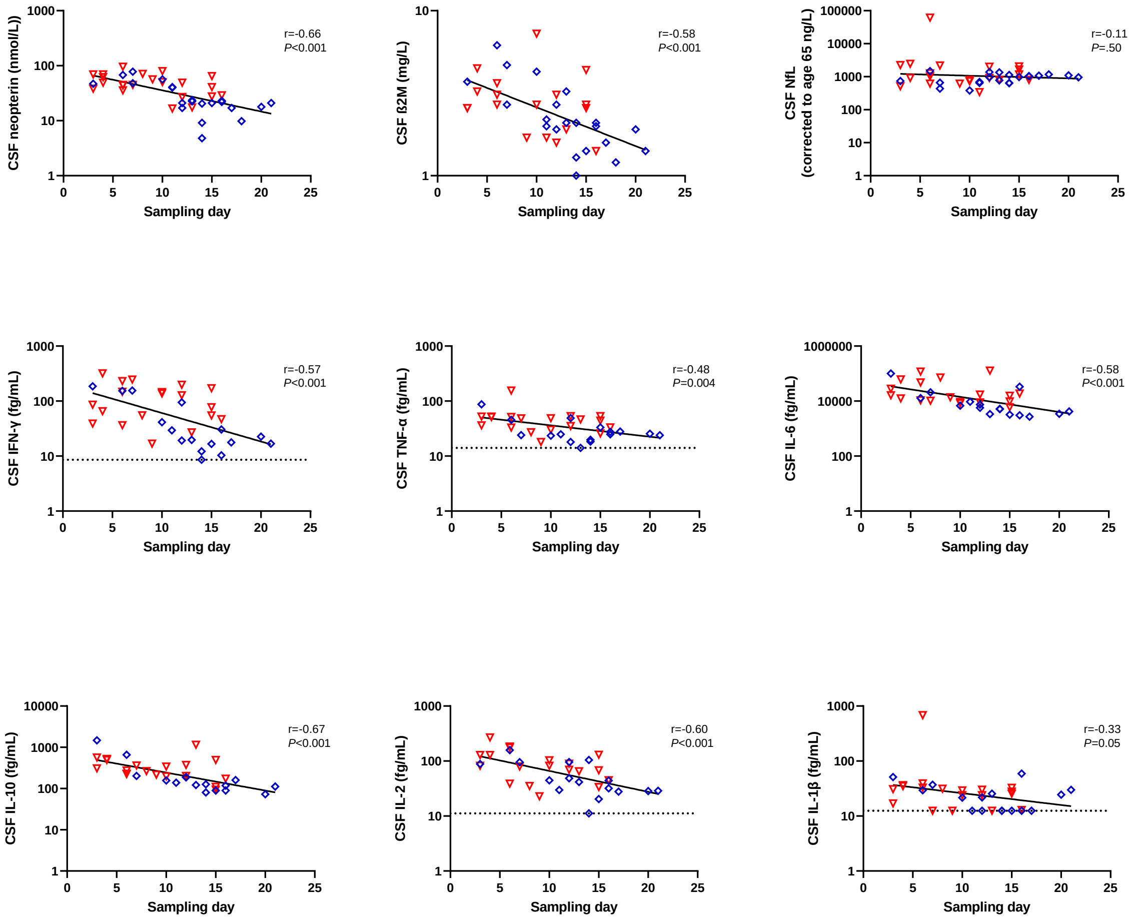

Neuroinflammatory CSF biomarker concentration trajectories in relation to time from any symptom onset to CSF sampling in COVID neurosymptomatic (red) and neuroasymptomatic (blue) patients. Correlations were estimated using Pearson correlation. All biomarkers, with the exception of CSF NfL, were significantly negatively correlated with the time from symptom onset to CSF sampling. Dotted lines represent assay limit of detection. CSF, cerebrospinal fluid; SARS-CoV-2, severe acute respiratory syndrome coronavirus-2; N-Ag, nucleocapsid antigen; S-Ag, spike antigen; WBC, white blood cell; IgG, immunoglobulin G; β2M, β2-microglobulin; NfL (65), neurofilament light protein (age-adjusted); IL, interleukin; TNF, tumor necrosis factor; IFN, interferon; CNS -, neuroasymptomatic; CNS +, neurosymptomatic.

© 2022 Edén A et al. *JAMA Network Open.*

eFigure 2. Inflammatory Serum Biomarker Profiles in COVID-19 Patient Groups and Controls

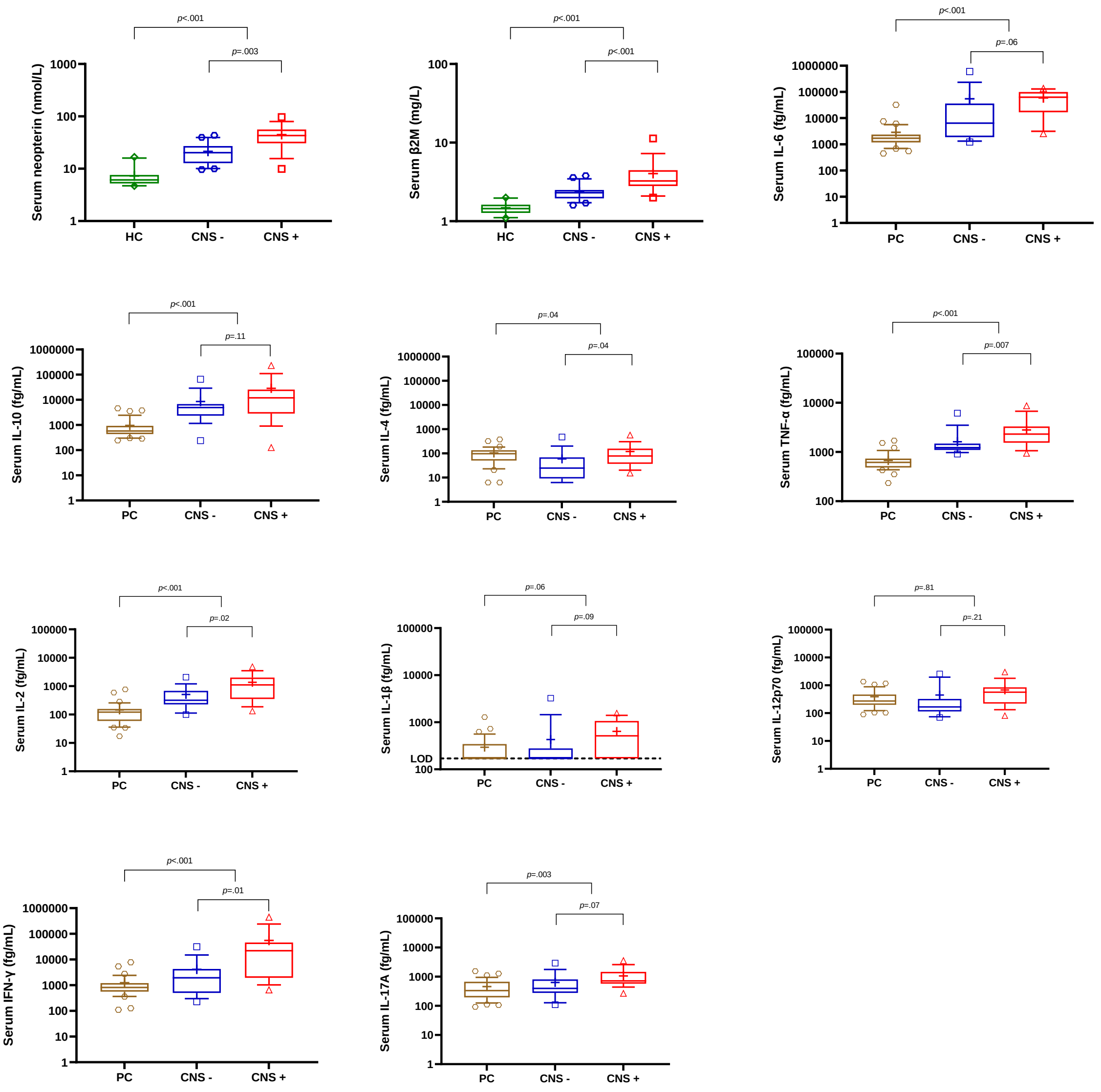

Profiles of serum biomarkers of inflammation/immune activation in the neuroasymptomatic (CNS-) and neurosymptomatic (CNS+) patient groups, "patient-control" (brown) and healthy control (green) groups. Boxes indicate the median and interquartile range, whiskers indicate the 10-90 percentiles, values above or below are drawn as individual points. Concentrations are shown as unadjusted values. Statistically significant differences are represented by *P* values and all statistical comparisons of biomarker concentrations between COVID patient groups were performed using ANCOVA adjusting for time from symptom onset to CSF sampling. Assay level of detection (LOD) is represented by a dashed line. SARS-CoV-2, severe acute respiratory syndrome coronavirus-2; β2M, β2-microglobulin; IL, interleukin; TNF, tumor necrosis factor; IFN, interferon; CNS -, neuroasymptomatic; CNS +, neurosymptomatic.

© 2022 Edén A et al. *JAMA Network Open.*

eReferences

1. Corman VM, Landt O, Kaiser M, et al. Detection of 2019 novel coronavirus (2019-nCoV) by real-time RT-PCR. *Euro Surveill.* 2020;25(3).
2. Pollock NR, Savage TJ, Wardell H, et al. Correlation of SARS-CoV-2 Nucleocapsid Antigen and RNA Concentrations in Nasopharyngeal Samples from Children and Adults Using an Ultrasensitive and Quantitative Antigen Assay. *Journal of clinical microbiology.* 2021;59(4).
3. Sigal GB, Novak T, Mathew A, et al. Measurement of SARS-CoV-2 antigens in plasma of pediatric patients with acute COVID-19 or MIS-C using an ultrasensitive and quantitative immunoassay. *medRxiv.* 2021:2021.2012.2008.21267502.
4. Wang H, Hogan CA, Verghese M, et al. SARS-CoV-2 Nucleocapsid Plasma Antigen for Diagnosis and Monitoring of COVID-19. *Clinical chemistry.* 2021.

© 2022 Edén A et al. *JAMA Network Open.*
